# Recovery from stroke: current concepts and future perspectives

**DOI:** 10.1186/s42466-020-00060-6

**Published:** 2020-06-16

**Authors:** Christian Grefkes, Gereon R. Fink

**Affiliations:** 1 Cognitive Neuroscience, Institute of Neuroscience and Medicine (INM-3), Research Centre Jülich, 52425 Jülich, Germany; 2 Medical Faculty, University of Cologne & Department of Neurology, University Hospital Cologne, 50924 Cologne, Germany

**Keywords:** Neurorehabilitation, Neuroimaging, Brain stimulation, TMS, Motor

## Abstract

Stroke is a leading cause of acquired, permanent disability worldwide. Although the treatment of acute stroke has been improved considerably, the majority of patients to date are left disabled with a considerable impact on functional independence and quality of life. As the absolute number of stroke survivors is likely to further increase due to the demographic changes in our aging societies, new strategies are needed in order to improve neurorehabilitation. The most critical driver of functional recovery post-stroke is neural reorganization. For developing novel, neurobiologically informed strategies to promote recovery of function, an improved understanding of the mechanisms enabling plasticity and recovery is mandatory.

This review provides a comprehensive survey of recent developments in the field of stroke recovery using neuroimaging and non-invasive brain stimulation. We discuss current concepts of how the brain reorganizes its functional architecture to overcome stroke-induced deficits, and also present evidence for maladaptive effects interfering with recovery. We demonstrate that the combination of neuroimaging and neurostimulation techniques allows a better understanding of how brain plasticity can be modulated to promote the reorganization of neural networks. Finally, neurotechnology-based treatment strategies allowing patient-tailored interventions to achieve enhanced treatment responses are discussed. The review also highlights important limitations of current models, and finally closes with possible solutions and future directions.

## Background

**Fig. 1:**
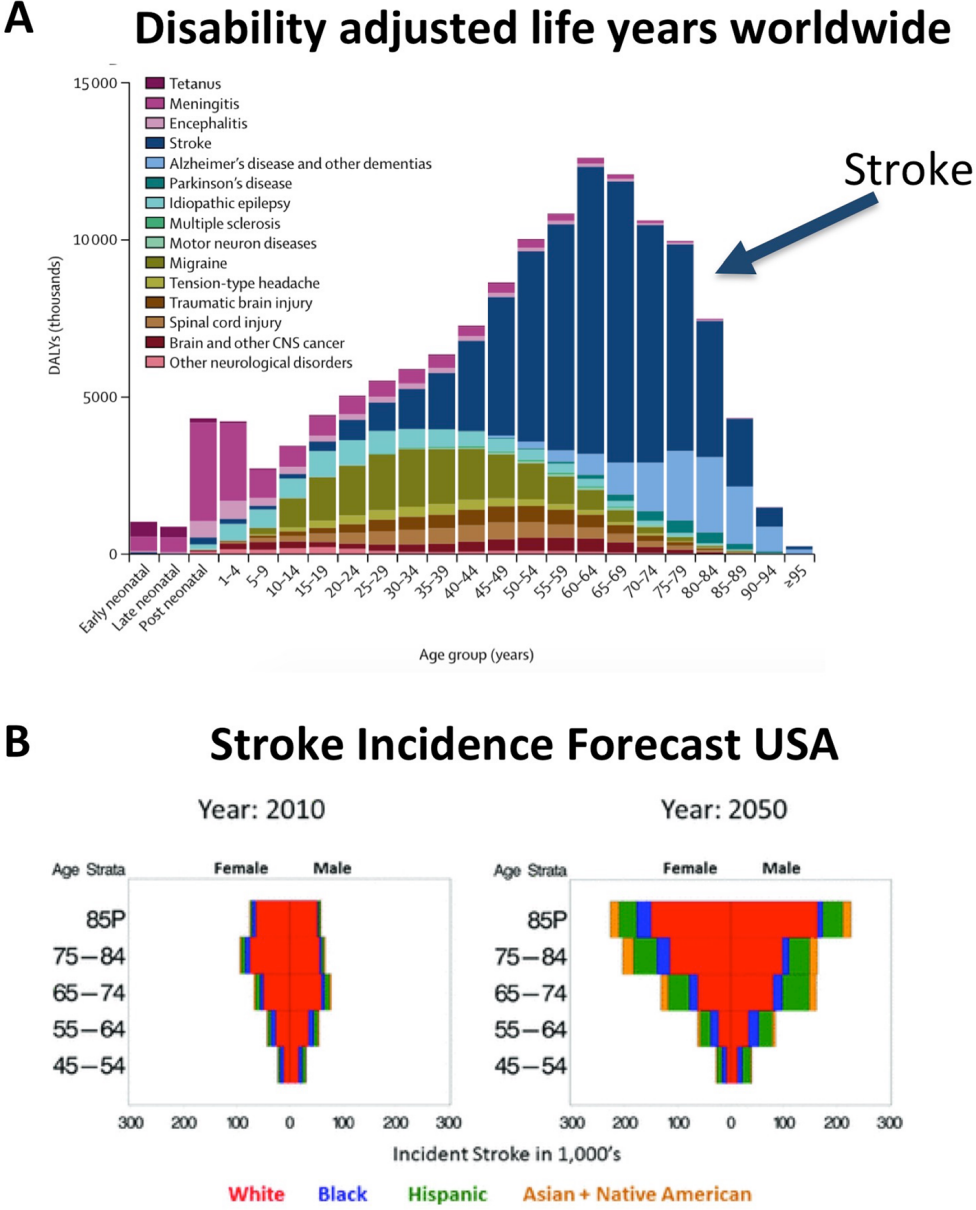
Stroke impact on society. **a** Disability-adjusted life years for 15 neurological disorder categories worldwide. With increasing age, stroke contributes the most to life years lost by death or disability. Shown are estimates for male persons; the graphs for females show similar percentages. Modified from the Global Burden of Disease Study group (2019,  [20]). **b** Projection of the distribution of incident stroke events in the US for the years 2010 and 2050, separated by ethnicity and age. Especially the proportion of very old patients (> 85 years) is expected to strongly increase over the next three decades. From [35] (with permission)

Stroke is the most common cause for acute hospitalization in neurology departments in high-income countries [13, 16, 60]. Like for other vascular diseases, stroke prevalence and incidence are strongly age-related. In Europe and the United States of America, the average age of all stroke patients is around 70 to 75 years (US National Center of Health Statistics, UK Stroke Association, German Medical Chamber). About two-thirds of all stroke patients are older than 65 years (e.g., CDC Stroke Statistics, Australian Stroke Foundation). The Global Burden of Disease (GBD) study group recently demonstrated that with higher age, stroke holds a paramount role concerning life years lost due to mortality or morbidity (disease-adjusted life years, DALY; Fig. 1a) [20]. Both age-standardized mortality and stroke prevalence rates have significantly decreased over the last three decades owing to better prevention of cardiovascular diseases in general and improvements in the acute stroke setting, e.g., specialized facilities (i.e., stroke units) and the development of recanalizing therapies, i.e., thrombolysis and thrombectomy. Nevertheless, absolute numbers of stroke deaths and DALY are still rising because of higher life expectancies and population growth in most countries [20]. In the next 30 years, these numbers are predicted to increase significantly [18]. In addition, demographic data suggest that in 2050, one out of three stroke patients will be 85 or older ([35]; Fig. 1b). Hence, there will be a greater need for higher capacities in stroke care especially for old and very old patients, as well as for enriched neurorehabilitation to improve stroke outcome in general.

While thrombolysis and thrombectomy are highly effective in reducing stroke-related morbidity and mortality [21, 66], only a relatively small minority of patients meet the criteria to obtain them [2, 45]. While the thrombolysis rate for single hospitals may reach up to 34% patients [17], numbers reported in larger regional registries including tens of thousands of patients are considerably smaller. For example, in 2018, approx.. 14.5% of the stroke patients in the German Northrhine area received thrombolysis (Medical Chamber Northrhine/Germany, Quality report 2018). For thrombectomy, approx. 5% of the patients were subjected to this treatment in 2018. Furthermore, even for patients having received thrombolysis or thrombectomy, the majority (> 50%) is left with a disabling neurological deficit, albeit significantly smaller than without treatment [21, 66].

Therefore, the development of new therapies that improve rehabilitation is warranted. To date, breakthroughs like those encountered in acute stroke treatment are missing. The reason is that we know much better what causes a stroke (i.e., a blood clot in a vessel or its rupture) than what causes recovery of function. Therefore, to promote recovery, it is necessary to understand the underlying (patho-)physiological processes.

### Recovery from stroke

The time after a stroke is often divided into phases. The Stroke Roundtable Consortium proposed to designate the first 24 h as the *hyperacute* phase, the first 7 days as the *acute* phase, the first 3 months as the *early sub-acute* phase, the months 4–6 as the *late sub-acute* phase, and from 6 months on as the *chronic* phase [3]. The rationale behind this differentiation is that recovery-related processes post-stroke are time-dependent. Already within hours after the onset of cerebral ischemia, a cascade of plasticity-enhancing mechanisms leads to dendritic growth, axonal sprouting, and the formation of new synapses [9, 39]. Furthermore, the most significant improvements occur in the first few weeks post-stroke, often reaching a relative plateau after 3 months with less significant recovery subsequently, especially concerning motor symptoms [40, 46]. After 6 months, spontaneous recovery is usually at its limit, leading to a more or less stable, i.e., chronic deficit. Nevertheless, with training or other interventions, improvements of some stroke-induced deficits can even be achieved in the chronic phase, primarily for more cognitive domains like language [14].

**Fig. 2:**
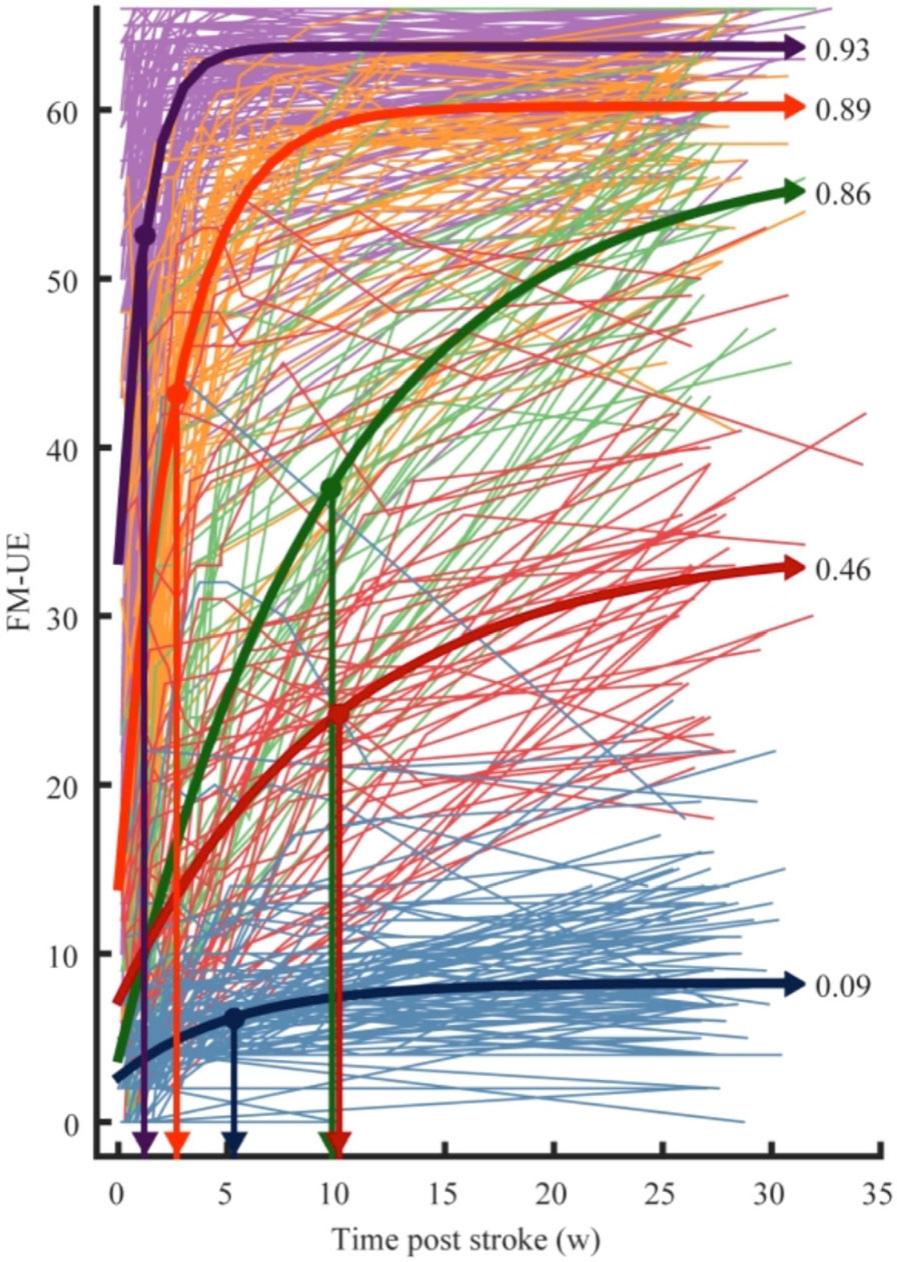
Motor recovery after stroke in a sample of *n* = 412 ischemic stroke patients based on the Fugl-Meyer upper extremity (FM-UE) score. Patients with mild initial deficits make on average better recovery than patients with severe deficits. Different colors represent different recovery subgroups based on a longitudinal mixture model. The numbers next to the recovery graphs represent the proportional recovery coefficient r_K,_ which denotes how much of the potential recovery has been achieved based on the FM-UE score. The downward arrows indicate the time constants τ_k_ in weeks, i.e., how fast patients recovered (here: reaching 1-e^− 1^ = 63.2% of total recovery). Of note, also initially severely affected patients (green curve) can achieve a good outcome with a relatively high recovery coefficient (r_k_ = 0.86) but a longer time constant (τ_k_ = 9.8 weeks) compared to the other subgroups. From Van der Vliet et al. [71]

While a distinct classification of post-stroke phases facilitates comparing the results reported by different studies, it carries the particular risk of considering functional recovery as a clear-cut sequence of phases rather than a continuous non-linear process. However, it appears very likely that recovery-associated processes 10 days post-stroke substantially differ from those 80 days post-stroke, although both are deemed to the same phase, i.e., the early subacute phase. Furthermore, recovery profiles greatly vary between subjects [71], with some patients recovering faster and better than others (Fig. 2), raising the question of whether the same processes underlie recovery for a given phase. Therefore, instead of using labels like ‘subacute’ or ‘chronic’ that are often implicitly used to indicate a particular potential for improvement, providing absolute numbers on time from stroke onset, e.g., weeks, besides further information about the level of impairment and stroke location, seems to be better suited to acknowledge the complex, non-linear nature of stroke recovery.

A rule of thumb in stroke recovery is that patients with mild deficits are more likely to make a good recovery than patients with initially more severe deficits (Fig. 2). The ‘proportional recovery rule’ assumes that patients can on average improve around 70% (+/− 15%) of their lost function within 3–6 months after stroke [50, 64, 79], with the lost function defined as being the hypothetical difference between normal function (e.g., a full score in a motor test) and the initial deficit of the patient. The proportional recovery rule is an interesting concept which assumes that recovery of function follows a fundamental neurobiological process that cannot be substantially influenced by whether a patient receives high- or low-intensity therapy [64]. It has, however, been criticized recently to be spuriously driven by mathematical coupling and ceiling effects, leading to over-estimations of proportional recovery relationships [31, 33, 71]. Besides, there seems to be a relevant number of patients who do not follow the proportional recovery rule (“non-fitters”) [31]. Especially patients with initially more severe deficits deviate from the proportional recovery rule, with a spectrum ranging from either showing almost no to very strong recovery (Fig. 2, green and blue lines, [71]). Some stroke patients with initially severe deficits like hemiplegia may even recover within the first 10 days [24], challenging current models of recovery with stringent phases. Applying advanced modeling approaches implementing Bayesian statistics revealed that across the entire spectrum of clinical impairment, the majority of stroke patients followed a combination of proportional-to-spared-function and constant recovery [5]. In contrast, only a minority of patients featured the “classical” proportional (to lost function) recovery rule [5]. In the same vein, Van der Vliet et al. [71] modeled longitudinal recovery profiles in a relatively large sample of patients (*n* = 412) and identified five subgroups that differed in how fast and well they recovered (Fig. 2). Although the neurobiological underpinnings underyling these sub-groups are yet to be explored, factors that are likely to contribute to different recovery rates include the amount of perilesional edema as well as ‘diaschisis’, i.e. remote effects on distant but structurally-functionally connected brain regions due to a lesion [11, 74].

From animal models, we have already learned that functional recovery is strongly associated with the formation of new synaptic connections. Mainly surviving neurons in peri-infarct tissue show an enlargement of their dendritic trees as well as sprouting of axons in order to form new connections with both local and distant brain areas [10]. Importantly, also axons of neurons in contralesional brain regions sprout and grow toward denervated tissue in both the ipsilesional and contralesional hemisphere as well as the brain stem and spinal cord. Such effects have been particularly observed in animals with more massive strokes [10]. Interestingly, some regions in the contralesional hemisphere can form aberrant connectivity that seems to hinder functional recovery, e.g., by transcallosal projections suppressing the cortical representation of the paretic limb due to maladaptive synaptic plasticity [38]. Therefore, a stroke induces different patterns of axonal sprouting, leading to both reparative and detrimental effects.

In summary, recovery from stroke seems - at least in part - to follow specific rules. However, it is still poorly understood why some patients recover and others do not. A better understanding of the neurobiology and causes of differential recovery profiles is, however, fundamental to design specific treatment regimes to improve the functional outcome after stroke. Although animal models provide valuable insights into the neurobiological processes associated with neural repair, major translational breakthroughs are missing in the field of neurorehabilitation. One important reason for this translational roadblock is the interspecies difference in structural and functional brain organization between rodents -which is the most often used animal model in preclinical stroke research- and humans. This also applies to the behavioral readouts, e.g., locomotion parameters in rodents versus unilateral hand motor performance in humans. Therefore, it is necessary to obtain a better understanding of how the complex cortico-cortical and cortico-subcortical networks of the *human* brain reorganize during recovery from a stroke-induced deficit.

### Imaging stroke recovery

**Fig. 3:**
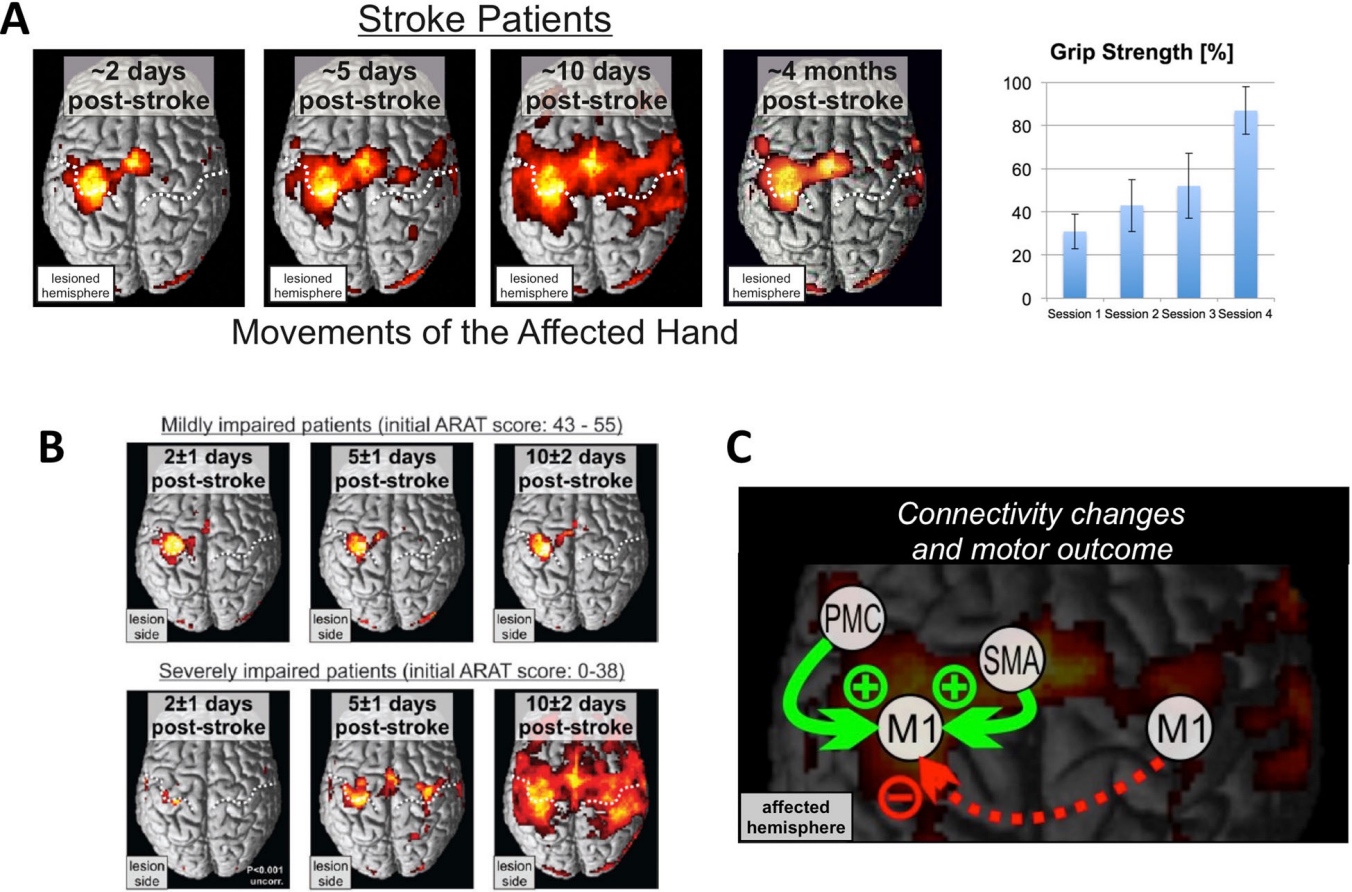
Neuroimaging of motor network reorganization after a first-ever stroke. **a** fMRI activity maps associated with movements of the paretic hand at different time points post-stroke. Recovery of hand motor function (here: maximum grip strength) is associated with fMRI signal increases early after stroke, the latter returning to levels observed in healthy controls with good functional recovery. From Rehme et al. [52, 53], with permission. **b** Impact of motor impairment and activity changes post-stroke. From Rehme et al. [52, 53], with permission. **c** Connectivity changes correlated with motor outcome. Green arrows: Increases indicate good functional motor outcome. Red arrow: Patients developing inhibitory influence from contralesional upon ipsilesional M1 activity feature poor functional motor outcome

Neuroimaging methods offer the unique opportunity to reveal the neural processes driving the recovery of function in patients non-invasively [23]. Especially functional magnetic resonance imaging (fMRI) has strongly extended our insights into the neural mechanisms underlying brain reorganization after stroke. A consistent finding across several fMRI studies in patients suffering from a motor stroke is that activity is altered not only in the lesioned hemisphere but likewise in the unaffected, i.e., contralesional hemisphere [12, 47, 51, 78]. For example, in contrast to healthy persons unilateral movements of the stroke-affected hand are typically associated with activity increases also in contralesional sensorimotor areas (Fig. 3a). A stronger recruitment of the contralesional hemisphere can be observed already within the first week post-stroke and is more likely to occur in patients suffering from more severe initial impairments (Fig. 3b [52, 53]).

In contrast, ipsilesional activity is usually attenuated in severely affected patients in the first few days post-stroke. Similar effects have also been reported for other functional systems, e.g., for the language system in patients with aphasia [59]. Importantly, in the motor system, first increases in brain activity in both ipsi- and contralesional areas correlate with functional recovery [52, 53]. However, longitudinal studies have shown that these activity increases represent a transient phenomenon in patients making good functional recovery 3 months later (Fig. 3a) [28, 78]. In contrast, patients with persistent deficits usually keep overactivity of the contralesional hemisphere, especially those with ipsilesional corticospinal tract lesions [76].

There is an ongoing discussion about the functional relevance of activity changes observed post-stroke [25, 67, 77]. For example, increases in contralesional activity might represent a mechanism supporting neural processing of the lesioned hemisphere. Alternatively, enhanced contralesional activity might result from transcallosal disinhibition, and hence, in turn, could even disturb coordinated neural processing within the lesioned hemisphere. In order to disentangle the role of a given area for the entire network, computational models of connectivity have been proven to be very useful [23]. Here, dynamic causal modelling (DCM) has been specifically designed to model the effective connectivity from fMRI time-series derived data [19]. Applying DCM to fMRI recorded while patients (10 ± 7 weeks post-stroke) performed movements with their stroke-affected hand revealed that the contralesional primary motor cortex (M1) exhibits an inhibitory influence on the activity of the ipsilesional M1 [26]. Of note, the strength of this inhibitory coupling correlated with the degree of motor impairment with more impaired patients featuring stronger inhibition. These data are compatible with a maladaptive role of contralesional M1 2–3 months post-stroke. Importantly, longitudinal data indicated that in the first few days after a stroke, contralesional M1 exerts a positive influence on ipsilesional M1 [52]. Correlating DCM coupling changes over time with motor outcome revealed that patients developing an inhibitory coupling were those with less favorable outcomes (Fig. 3c). In contrast, higher increases in coupling from ipsilesional premotor areas to ipsilesional M1 were associated with good motor outcomes [52, 53]. Therefore, a good motor outcome after stroke is linked to the reinstatement of a network configuration lateralized to the ipsilesional hemisphere, hence resembling the situation observed in healthy subjects [24, 75]. However, in well-recovered patients, the contralesional hemisphere may also exert a supportive influence. For example, Pool et al. [49] showed that in patients 1.5 years (17.5 ± 9.4 months) post-stroke, the contralesional superior posterior parietal cortex exerts a positive influence on ipsilesional M1 activity the more demanding a motor task gets for the paretic hand. As this supporting influence is absent in healthy controls, it may be concluded that the posterior parietal cortex contributes to motor recovery after stroke [49].

In summary, analyses of fMRI activity and connectivity revealed that a focal ischemic lesion affects brain areas not only in the vicinity but also at remote locations in both hemispheres. Recovery from stroke-induced motor impairments is closely related to network changes, with contralesional areas also influencing the motor performance of the paretic hand. Similar findings have also been reported for other functional domains of the brain, e.g., for the language system in patients recovering from aphasia [65].

### Interference with stroke recovery

**Fig. 4:**
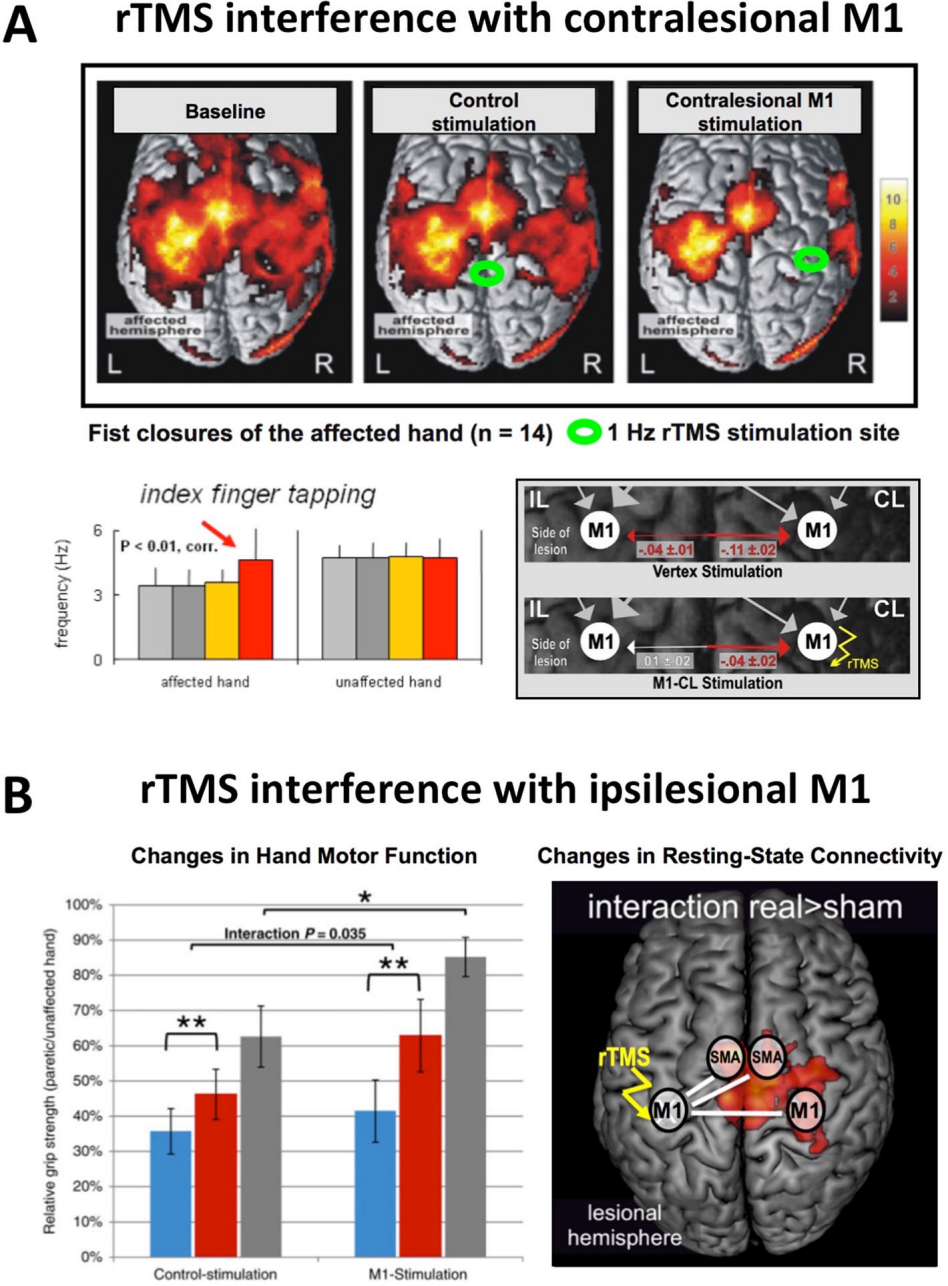
Shaping brain networks post-stroke using non-invasive brain stimulation. **a** Application of 1 Hz rTMS to suppress contralesional M1 activity leads to normalization of brain activity associated with movements of the stroke-affected hand. Connectivity analyses reveal that inhibitory influences originating from M1 of the unaffected hemisphere disappear after 1 Hz treatment compared to baseline measurement or sham stimulation. From Grefkes et al. [27], with permissions. **b** Application of iTBS rTMS to enhance ipsilesional M1 activity in the first weeks after stroke improves recovery of grip strengths (blue: baseline measurement; red: post-intervention measurement; grey: follow-up measurements 3 months later). At the neural level, patients having received iTBS over M1 feature more robust resting-state functional connectivity of the stimulated motor cortex with ipsi- and contralesional sensorimotor areas. From Volz et al. [72], with permission

A strong driver of neural plasticity and hence reorganization after stroke is training. Classical training-based interventions such as physical, occupational, or language therapy as well as novel multimodal approaches like, e.g., mirror therapy or music-based therapy, have all been shown to enhance functional recovery, albeit to variable degrees (see, e.g., [22, 30]). Given the intimate relationship between motor performance and neural activity, a promising alternative for improving stroke-induced deficits is to *directly* manipulate brain activity. Non-invasive brain stimulation techniques like transcranial magnetic stimulation (TMS) or transcranial direct current stimulation (TDCS) can be used to modulate neural plasticity [41, 58, 62]. Importantly, TMS- or TDCS-induced changes of regional activity have been demonstrated to propagate towards interconnected brain regions, thereby influencing activity within the entire network of the stimulated node [43, 63]. Therefore, these approaches seem to be useful when aiming at correcting pathological network configurations as encountered after stroke. For example, when suppressing the activity of contralesional M1 using inhibitory 1 Hz repetitive TMS (rTMS) in patients 2 ± 1 months post stroke, fMRI over-activity was not only reduced in the stimulated region but also the ipsilesional hemisphere (Fig. 4a). As a result, this yielded a more lateralized activation pattern compared to the baseline condition or sham/control-stimulation [47]. Importantly, the “normalization” of activity was associated with an increase in motor performance of the paretic hand. Connectivity analyses furthermore revealed that this “normalization” of activity was linked to an attenuation of abnormal inhibitory influences from contralesional M1 (Fig. 4a), especially in patients showing the most significant motor improvement [27].

Motor improvements upon TMS-evoked inactivation of contralesional M1 can be already observed in the first week post-stroke in patients with mild motor deficits [73]. Of note, this effect was absent when interfering with contralesional M1 3 months later, when patients recovered to performance levels as observed in healthy controls. Likewise, another study of us [68] showed that the functional role of contralesional areas change during functional recovery. While contralesional anterior parietal cortex exerted a negative effect on motor performance of the stroke-affected hand in the first week after stroke (3.6 ± 1.7 days) as well as 3 months later, contralesional dorsal premotor cortex developed a supporting influence during recovery. Similar supportive effects of the contralesional dorsal premotor cortex were also observed in other fMRI and TMS studies [4, 37]. Furthermore, a more influential role of the dorsal premotor cortex for hand movements has also been observed in physiological aging [69], implying a general neural mechanism to support hand motor function. In summary, the roles of contralesional areas in the recovery of motor function post-stroke are region-specific and time-sensitive, which needs to be considered when using non-invasive brain stimulation to improve the functional outcome after stroke.

Also, activity changes of ipsilesional brain regions induced by non-invasive brain stimulation techniques have been linked to post-stroke recovery [52, 53]. Particularly stimulation of ipsilesional M1 using excitability-enhancing rTMS protocols was shown to interfere with motor performance and recovery. For example, Ameli and colleagues [1] used a 10 Hz rTMS protocol to increase ipsilesional M1 excitability in patients with a wide range of time post-stroke (1–88 weeks, average, 22.2 ± 26.0 weeks). The authors found that only patients with subcortical lesions responded with an improved motor performance of the stroke-affected hand, which was accompanied by a normalization of fMRI activity. In contrast, patients with cortical infarcts did not improve after the intervention, and also brain activity remained abnormal. Therefore, the lesion extent seems to be a critical variable linked to the response following ipsilesional M1 stimulation.

Other critical variables for an rTMS response seem to be time from stroke onset and treatment dose. For example, when applying intermittent theta-burst stimulation (iTBS; an rTMS protocol known to predominantly increase excitability [29, 44]) to ipsilesional M1 of patients suffering from chronic motor deficits (> 12 months; average 5.2 ± 5.4 years), a single rTMS block had no behavioral effect at the group level, despite increases in ipsilesional M1 excitability [15]. Although the connectivity strength of the stimulated ipsilesional M1 correlated with interindividual differences in behavioral after-effects, the general response to rTMS remained weak. A reason for this might be that plasticity induction in a reorganized brain years after stroke is difficult, with some patients improving but others deteriorating. The situation might be different early after stroke when endogenous plasticity is already upregulated [14]. Furthermore, the early post-acute stroke phase is characterized by a loss of ipsilesional activity [24, 52, 53]. Hence, it seems reasonable to assume that inducing activity in ipsilesional M1 early after stroke is beneficial for stroke recovery, especially when applied repetitively. Therefore, Volz et al. [72] employed rTMS using an iTBS protocol combined with motor training in patients recovering from a first-ever stroke. Stimulation was administered within the first 2 weeks post-stroke (on average: 7.3 ± 3.6 days) for five consecutive days and compared to a control group receiving sham stimulation. After the intervention week, the patients having received M1 stimulation featured better improvements of grip strength of their paretic hand, compared to the control group (Fig. 4b). This difference was still detectable at a follow-up measurement 3 months later. The connectivity analysis revealed that M1-stimulated patients showed higher ipsilesional M1 connectivity with ipsilesional as well as contralesional areas, compared to the control group (Fig. 4b). Therefore, pairing iTBS with motor training early after stroke seems to promote motor recovery by enhancing motor network connectivity [72]. The clinical impact of this study is currently tested in a phase-3 randomized controlled trial (TheSiReS trial [32];.

### Open questions and future perspective

Functional neuroimaging has substantially advanced our understanding of the neural mechanisms engaged in the recovery of function after a stroke and brain stimulation-induced improvements. Also, interindividual variability concerning recovery or treatment response can be linked to brain imaging markers such as connectivity. Recent developments in the analysis of fMRI data such as dynamic functional connectivity now even allow investigating the effects of stroke on temporal network dynamics and their link to motor impairment [6]. However, despite all these advances, we are still far away from a personalized approach that considers the individual network pathology in order to precisely correct dysconnectivity of specific network nodes. First attempts have already been made, e.g., by using multivariate machine learning techniques to predict motor impairment [54] or motor outcome [55] based on fMRI data acquired in the first week after stroke. However, it remains debatable whether individual predictions can yet rely on a single MRI network readout to achieve diagnostic accuracy. For example, Siegel and colleagues [61] demonstrated in a relatively large sample of patients (*n* = 100) 1–2 weeks post-stroke that around 20% of the variance observed in post-stroke motor symptoms can be explained by resting-state functional connectivity, while lesion topography predicts about 45% of motor deficits. Hence, despite these significant predictions, the overall prediction performance is still far from being accurate enough to be reliably used at a single patient level.

The critical question is whether (f)MRI is the method of choice to make individual predictions of outcome and recovery after stroke. Apart from the fact that fMRI measurements are susceptible to head motion - a problem frequently encountered in patient populations -, especially stroke patients often feature small or large vessel disease interfering with blood flow. Therefore, for a relevant proportion of patients, no valid fMRI signal can be obtained. Furthermore, especially severely affected patients are underrepresented in fMRI research. A possible reason for this underrepresentation is that apart from problems to obtain informed consent for study participation, the logistic and medical effort to examine these patients is relatively high. Therefore, most of our ideas and conclusion related to post-stroke recovery are biased towards patients with relatively mild to moderate deficits, and it remains questionable whether supporting or maladaptive neural mechanisms also hold for patients with extended cerebral infarcts.

Recently, studies using electroencephalography (EEG) have experienced a renaissance due to improvements in recording and analyzing techniques [7, 56]. For example, Bonstrup et al. [7] recorded high-density EEG while hemiparetic patients within 5 days post-stroke performed an isometric visually guided whole-hand grip task with their paretic hand. The authors found that the re-emergence of low-frequency oscillations during movement preparation coincided with hand motor recovery with more robust increases in patients making a better recovery. Hence, this time-sensitive parameter might serve as a novel biomarker of recovered brain function that could, in the next step, be targeted in therapeutic approaches, e.g., using non-invasive brain stimulation.

Likewise, the combination of transcranial magnetic stimulation (TMS) with high-density EEG represents a non-invasive perturb-and-measure approach that simultaneously informs about local neuronal states as well as signal propagation at the functional network level [36]. Therefore, this approach holds the potential to serve as a non-invasive network readout in individual patients that can be performed at the bedside in severely affected patients [57]. Tscherpel et al. [70] reported that measures of TMS-evoked EEG responses in the first days after stroke are closely related to the initial motor deficit and the amount of clinical recovery after more than 3 months post-stroke. Importantly, also in patients that were indistinguishable based on their phenotypical/clinical presentation (i.e. no residual arm function, no peripheral motor-evoked potential upon “conventional” TMS), TMS-EEG was able to disclose differential response patterns that correlated with subsequent recovery. This highlights the enormous potential of this technique to serve as a novel readout of the functional reserve of the motor network. Also, other studies found close relationships between TMS-EEG parameters and stroke symptoms or outcomes [8, 34, 42, 48]. However, whether or not this technique allows true (i.e., out-of-sample) predictions for recovery remains to be elucidated in future studies with large sample sizes.

## Conclusions

Given the demographic change of our aging society and better acute stroke treatment opportunities leading to improved survival rates of stroke patients, the absolute number of patients with stroke-induced deficits is likely to increase over the next decades. Hence, novel treatment strategies post-stroke are needed to reduce stroke-induced morbidity and to increase the patient’s and caregiver’s quality of life. Functional neuroimaging, as well as non-invasive brain stimulation techniques, have significantly advanced our understanding of stroke-induced reorganization of brain networks. Evidence is accumulating that network effects distant to the lesion contribute to the motor deficit and recovery thereof significantly. That these effects are time- and region-dependent impacts future strategies to shape network reorganization through brain stimulation techniques. Given the large variability in network responses following a stroke, individual network readouts and/or multivariate decoding techniques seem mandatory for the stratification of patients to achieve an optimal therapeutic response.

## Data Availability

Not applicable.

## Abbreviations

BOLD: Blood oxygenation dependent
DALY: Disease adjusted life years
dPMC: Dorsal premotor cortex
GBD: Global burden of Disease
M1: Primary motor cortex
fMRI: Functional magnetic resonance imaging
MRI: Magnetic resonance imaging
TDCS: Transcranial direct current stimulation
TMS: Transcranial magnetic stimulation
TMS-EEG: Transcranial magnetic stimulation combined with electroencephalography
